# Disease-induced changes in bacterial and fungal communities from plant below- and aboveground compartments

**DOI:** 10.1007/s00253-024-13150-1

**Published:** 2024-04-30

**Authors:** Mingfeng Cao, Songqing Huang, Jingjing Li, Xiaoming Zhang, Yi Zhu, Jingzhao Sun, Li Zhu, Yong Deng, Jianqiang Xu, Zhihua Zhang, Qiang Li, Jixiang Ai, Tian Xie, Hengli Li, Huaqun Yin, Wuyuan Kong, Yabing Gu

**Affiliations:** 1 Changde Tobacco Company of Hunan Province, Changde, China; 2 School of Minerals Processing and Bioengineering, Central South University, Changsha, China; 3 Technology Center of China Tobacco Fujian Company, Xiamen, China

**Keywords:** *Potato virus Y* disease, Rhizosphere, Endophytes, Microbial community, Microbial network

## Abstract

**Abstract:**

The plant microbes are an integral part of the host and play fundamental roles in plant growth and health. There is evidence indicating that plants have the ability to attract beneficial microorganisms through their roots in order to defend against pathogens. However, the mechanisms of plant microbial community assembly from below- to aboveground compartments under pathogen infection remain unclear. In this study, we investigated the bacterial and fungal communities in bulk soil, rhizosphere soil, root, stem, and leaf of both healthy and infected (*Potato virus Y* disease, PVY) plants. The results indicated that bacterial and fungal communities showed different recruitment strategies in plant organs. The number and abundance of shared bacterial ASVs between bulk and rhizosphere soils decreased with ascending migration from below- to aboveground compartments, while the number and abundance of fungal ASVs showed no obvious changes. Field type, plant compartments, and PVY infection all affected the diversity and structures of microbial community, with stronger effects observed in the bacterial community than the fungal community. Furthermore, PVY infection, rhizosphere soil pH, and water content (WC) contributed more to the assembly of the bacterial community than the fungal community. The analysis of microbial networks revealed that the bacterial communities were more sensitive to PVY infection than the fungal communities, as evidenced by the lower network stability of the bacterial community, which was characterized by a higher proportion of positive edges. PVY infection further increased the bacterial network stability and decreased the fungal network stability. These findings advance our understanding of how microbes respond to pathogen infections and provide a rationale and theoretical basis for biocontrol technology in promoting sustainable agriculture.

**Key points:**

*• Different recruitment strategies between plant bacterial and fungal communities.*

*• Bacterial community was more sensitive to PVY infection than fungal community.*

*• pH and WC drove the microbial community assembly under PVY infection.*

**Supplementary Information:**

The online version contains supplementary material available at 10.1007/s00253-024-13150-1.

## Introduction

*Potato virus Y* (PVY) is the type member of the genus *Potyvirus*, a major plant virus pathogen that causes significant economic losses worldwide (Faurez et al. 2012). Researchers have found that PVY infection can lead to foliar and/or tuber disease, with symptoms varying based on the virus strain, host growth stage and susceptibility, and environmental conditions (Fox et al. 2017). Infection non-persistently of PVY by aphids (e.g., *Myzus persicae*) has been found to be a major method between plants through infected sap (Deja-Sikora et al. 2019). Recently, research has extensively focused on the evolutionary history, functional dissection, infection sources (Coutts and Jones 2015), transmission modes (da Silva et al. 2020), and plant resistance (Petrov et al. 2015). However, infected residues in seeds and plant tissue in soil are the primary infection sources for virus diseases, and the above practices are ineffective in preventing the spread of PVY infection. Therefore, it is now necessary to develop potentially successful strategies to manage the virus from its primary infection sources (e.g., soil) and prevent its spread in plants. Research about the interactions between crop plants and diverse microorganisms provides a potential method through microbiological agents.

Plant growth and physiology are influenced by the microbiome, including viruses, bacteria, fungi, insects, and other invertebrates. These diverse biotic interactions (involving antagonistic, protective, exclusive, or symbiont effects) among tripartite members are of particular interest to plant crops due to their impacts on crop production (Deja-Sikora et al. 2019). It is documented that the extensive microflora presenting in the phyllosphere, endosphere, and rhizosphere forms influence plant growth, soil fertility, and disease (Etesami and Adl 2020). Bacteria and fungi are two major groups that lead to many biological effects through antibiotics, signaling molecules, physiochemical environment modulation, chemotaxis, cooperative metabolism, protein secretion, and even gene transfer (Frey-Klett et al. 2011). Various biological strategies based on biotic interactions are carried out to control plant diseases caused by pathogenic microorganisms, such as the application of microbial agents. For decades, huge studies have described the diversity of microbiomes associated with agricultural crop plants, paving the way for the use of microbes as biofertilizers and biopesticides (Lemanceau et al. 2017). For example, *Bacillus amyloliquefaciens* FZB42 is a commercially available bacterial strain that could produce bacillomycin D to resist the fungus *Fusarium graminearum* that infects wheat and barley (Gu et al. 2017). The wheat microbiota has been shown to reduce the virulence of the plant pathogenic fungus *Fusarium graminearum* by altering histone acetylation (Chen et al. 2018). Although many microbial agents present in agricultural ecology have been applied to control microbial diseases, the colonization of functional microorganisms is the key limiting factor. Understanding the complex biotic effects of the plant microbiome provides new insights for the development of more efficient and stable microbial agents in agricultural production.

Microbial communities in plant rhizosphere and endosphere habitats are both important for controlling PVY infection and transmission, respectively. Pathogens of PVY primarily exist in the soil, and their infections can be modulated by rhizosphere microbes. Some plant growth–promoting rhizosphere microorganisms have the ability to synthesize various organic compounds, phytohormones, siderophores, and lytic and antioxidant enzymes. Additionally, they can enhance the stress-responsive ability of plants to remove plant pathogens from the soil (Hashem et al. 2017). Plant species, distinct rhizosphere environments (e.g., soil properties), and exudate blends were reported to be the main factors shaping the microbial assembly around plant roots (Berg et al. 2021). Endophytes are microbes that inhabit plant tissues and play crucial roles in plant growth, health, and resistance (Hardoim et al. 2015). They are classified into three types according to ecological functions: (i) The first group is called commensal endophytes, which show no apparent effects on plant performance. (ii) The second group provides beneficial effects for plants, such as plant growth promotion and protection against invading pathogens. (iii) The third group consists of latent pathogens. Endophytes are usually transmitted vertically through seeds and proliferate under local conditions inside the plant. Interestingly, interactions between endophytes and host plants can also affect rhizosphere microorganisms. Previous studies have shown that soil microbial activity can also be inhibited by endophyte infection of aboveground plants (Buyer et al. 2011; Tong et al. 2017).

Researchers have come to understand the importance of the effects of pathogen infections on rhizosphere and endophyte microbial communities. However, the ascending migration pattern of microbial communities under disease infection remains unclear. We hypothesized that bacterial and fungal communities would exhibit different responses to PVY infection, based on variations in body size, diversity, dispersal potential, ecological function, and correlation with the host and other microorganisms. In addition, bacterial communities may be more susceptible to infection than fungal communities. To test the hypotheses, we investigated the ascending migration pattern of microbes from below- to aboveground (both bacterial and fungal), explored taxonomic differences between healthy and diseased plants using amplicon sequencing, and compared microbial networks of healthy and infected plants.

## Material and methods

### Sampling

All samples were collected from the main tobacco production fields in Changde (29°13′30″–29°59′19″N, 110°28′40″–110°58′30″E), Hunan province, China. The two sites in this study were flooded fields (F) and upland paddy fields (P), respectively. The tobacco cultivars were the same at both sites, and planted using the same agronomic practices. Bulk soil, rhizosphere soil, and plant tissues (including root, stem, and leaf) were sampled in August 2022 from both fields. In each field, tobacco plants that showed significant height reduction and venous necrosis symptoms were classified as PVY-infected plants, while plants showing no significant symptoms were classified as healthy plants (Latorre et al. 1984). Six replicates of healthy and infected plants were collected from each field. Bulk soil was collected 20 cm away from the root, and rhizosphere soil was collected from soil adhering to the root by hand shaking. All samples were transported to the laboratory on dry ice.

Then, soil samples were divided into two parts: one part was stored at − 80 °C for microbial experiments, and the other part was sent to the School of Resources and Environment at Southwest University for soil properties measurement. The pH value, water content (WC), organic matter (OM), total nitrogen (TN), alkali hydrolysable nitrogen (AHN), total phosphorus (TP), available phosphorus (AP), total potassium (TK), and available potassium (AK) were tested. Plant tissue samples were washed with plenty of water to remove soil and dust from the tissue surface. Then, plant tissues (about 5g) were successively immersed in 70% ethanol for 10 min, 5.25% sodium hypochlorite solution for 5 min, and 70% ethanol for 1 min, and finally washed with sterile water (Gao et al. 2021; Liu et al. 2021). Treated tissues were ground with liquid nitrogen in a sterile mortar and then stored at − 80 °C for further microbial experiments.

Overall, there were 18 treatments in this study: nine treatments in flooded fields, including bulk soil (PC), rhizosphere soil (PZ: PHZ for healthy plants and PIZ for infected plants), plant root (PR: PHR and PIR), stem (PS: PHS and PIS), and leaf (PL: PHL and PIL), and nine treatments in paddy fields, including FC, FZ (FHZ for healthy plants and FIZ for infected plants), FR (FHR and FIR), FS (FHS and FIS), and FL (FHL and FIL).

## High-throughput sequencing and analysis

Total DNA was extracted from the soil and plant samples using the FastDNA SPIN Kit for Soil, following the manufacturer’s instructions. The primer 799F (5′-AACMGGATTAGATACCCKG-3′)/1115R (5′-AGGGTTGCGCTCGTTG-3′) was used to amplify the V5-V7 region of the bacterial 16S rRNA gene (Kembel et al. 2014; Deng et al. 2021), and primer fITS7/ITS4 was used to amplify the fungal ITS2 region (de Vries et al. 2018a). Sequencing was performed using the Illumina Hiseq2500 platform at MEGIGENE Biotechnology Co., Ltd. (Guangzhou, China). The raw data for bacteria and fungi were all uploaded to the NCBI database in the projects of PRJNA946037 and PRJNA946055.

The 16S rRNA and ITS sequences were processed using QIIME2 platform (2020.6) with default parameters (Zhang et al. 2022). First, primers from reads were removed to obtain clean sequences. Then, DADA2 was used to generate feature tables based on the clean sequences (Callahan et al. 2016). Finally, taxonomic assignment was conducted according to the SILVA reference database and the UNITE database for bacteria and fungi (Kõljalg et al. 2005; Quast et al. 2012), respectively. Singlet reads; bacterial amplicon sequence variants (ASVs) classified as chloroplast, mitochondrion, or Viridiplantae; and fungal ASVs classified as plant or protist were removed. The feature table and taxonomic table for bacteria and fungi were used for downstream analysis.

## Network analysis

In order to understand how microbial communities changed under PVY infection, four co-occurrence networks (HB: bacterial network for healthy plants, IB: bacterial network for infected plants, HF: fungal network for healthy plants, IB: fungal network for infected plants) were constructed using SparCC (Friedman and Alm 2012) by the *SpiecEasi* package in R (version 4.0.0) and visualized in Gephi (version 0.9.2). ASVs presenting in 1/6 of bacterial samples and 1/8 of fungal samples were used to construct networks with default parameters. SparCC was used to calculate correlations between ASVs with 100 permutations, and corrections with |coefficient|> 0.3 and *p* < 0.05 were incorporated into the network. Each node indicates a specific ASV, and each edge represents a significant correlation between ASVs. Topological characteristics of the network, such as the number of nodes and edges, proportion of positive edges, average degree, average path length, density, clustering coefficient, betweenness centralization, degree centralization, and modularity, were calculated to characterize the network complexity and stability (Hernandez et al. 2021) using *igraph* package. Based on the within-module connectivity (*Zi*) and among-module connectivity (*Pi*), the network nodes were identified as peripherals (*Zi* < 2.5 and *Pi* < 0.62), network hubs (*Zi* ≥ 2.5 and *Pi* ≥ 0.62), module hubs (*Zi* ≥ 2.5 and Pi < 0.62), and connectors (*Zi* < 2.5 and *Pi* ≥ 0.62) (Strogatz 2001). Network hubs, module hubs, and connectors were keystone species of networks (Shi et al. 2016).

## Data analysis

Alpha diversity indexes, including the Shannon index (H), species richness (S), and Pielou’s evenness (J), were calculated using the *vegan* package (Oksanen et al. 2007) in R (version 4.0.0). Differences between treatments (different compartments, healthy/infected plants, different field types) were tested using analysis of variance (ANOVA). Principal coordinates analysis (PCoA) based on Bray–Curtis distance was calculated and visualized using the *ggplot2* package. Permutational multivariate analysis of variance (PERMANOVA) statistical tests were performed to determine the effects of different factors on microbial community diversity by “*adonis*” in *vegan* R package, with 999 permutations. PERMANOVA was also used to test the effects of different factors on community structures. Three different complementary non-parametric analyses were used to test microbial community dissimilarity (Zhou et al. 2011), including an analysis of similarity (ANOSIM) (Clarke 1993), a multiresponse permutation procedure (MRPP), and a permutational multivariate analysis of variance (Adonis) (Anderson 2001). The Venn diagram was plotted to exhibit the ASV distribution using the *VennDiagram* R package. Spearman’s correlation analysis between soil properties and microbial genera and orders was conducted and visualized in a heatmap using the *vegan* and *pheatmap* R packages. Partial least squares path model (PLS-PM) was performed to decouple the effects of soil properties and PVY on microbial communities using the *plspm* R package (Latan et al. 2017; Jiang et al. 2021b). Comparison of bacterial composition at the genus level and fungal composition at the order level was conducted using Student’s *t*-test in STAMP (Parks et al. 2014; Gu et al. 2022a).

## Results

### Soil physical and chemical properties in flooded and upland paddy fields

Field and rhizosphere soil physical and chemical properties showed significant differences between flooded and upland paddy fields (Table 1). WC, pH, AHN, and TK were significantly higher in flooded fields than in upland paddy fields (ANOVA, *p* < 0.05). Additionally, soil properties also changed significantly in bulk soil, healthy plant rhizosphere soil, and infected plant rhizosphere soil. In flooded fields, soil physical and chemical properties were higher in rhizosphere soil than in bulk soil, but there was no obvious difference between healthy and infected plant rhizosphere soil. In upland paddy fields, the content of AK was significantly higher in rhizosphere soil than in bulk soil, and TP and AP were obviously higher in infected plant rhizosphere soil than in bulk soil. These results suggested that field type and plant were major factors in changing soil properties, and plant health state also played a regulatory role in soil properties.

**Table 1:** ANOVA of rhizosphere soil physical and chemical properties among treatments based on Turkey test. Data are means ± standard errors. Different letters indicate statistically significant differences among treatments (*p* < 0.05). *WC*, water content (%); *OM*, organic matter (g/kg); *TN*, total nitrogen (g/kg); *AHN*, alkali hydrolysable nitrogen (mg/kg); *TP*, total phosphorus (g/kg); *AP*, available phosphorus (mg/kg); *TK*, total potassium (g/kg); *AK*, available potassium (mg/kg)

Treatment	FC	FH	FI	PC	PH	PI
WC	16.59 ± 2.171b	20.84 ± 0.725a	20.71 ± 0.812a	11.95 ± 2.824c	13.88 ± 2.032c	13.79 ± 2.392c
pH	6.78 ± 0.941a	7.18 ± 0.542a	7.12 ± 0.293a	5.88 ± 0.535b	5.85 ± 0.554b	6.083 ± 0.349b
OM	28.00 ± 14.823ab	29.69 ± 10.623ab	32.84 ± 11.240a	19.14 ± 7.468b	21.24 ± 6.191b	23.15 ± 3.013ab
TN	1.79 ± 1.016ab	1.83 ± 0.732ab	2.28 ± 0.807a	1.37 ± 0.563b	1.43 ± 0.526b	1.61 ± 0.244ab
AHN	129.04 ± 63.43ab	126.32 ± 37.18ab	152.24 ± 57.50a	93.89 ± 39.38b	95.23 ± 33.51b	99.45 ± 8.49b
TP	0.74 ± 0.268bc	1.20 ± 0.411ab	1.41 ± 0.865a	0.58 ± 0.089c	0.89 ± 0.345abc	1.15 ± 0.378ab
AP	40.96 ± 34.47bc	109.51 ± 74.22a	88.23 ± 54.19ab	22.51 ± 10.39c	75.06 ± 50.01abc	121.8 ± 47.38a
TK	20.78 ± 4.414a	19.15 ± 5.03ab	22.68 ± 5.088a	13.11 ± 1.048c	13.42 ± 1.286c	15.33 ± 4.107bc
AK	529.2 ± 198.4bc	904.2 ± 377.3ab	900.0 ± 277.9ab	352.7 ± 156.3c	933.3 ± 560.7a	1004.2 ± 305.5a

### PVY affected the plant microbial community diversities

In total, 32,339 bacterial ASVs and 3611 fungal ASVs were observed from 108 samples. To determine the dimensions in which factors shape the plant microbiome, the relative contribution of multiple factors in terms of compartment (bulk soil, rhizosphere soil, root, stem, and leaf), PVY (health or infection), and field type (flooded field or paddy field) was assessed in shaping bacterial and fungal communities. Microbial community diversity indexes, including Shannon index (H) and Pielou’s evennesses (J), were calculated (Table S1, S2). ANOVA analysis suggested that the bacterial community diversities (H and J) in rhizosphere soil were significantly higher than that in the endophytic bacterial community diversities, while the endophytic bacterial community diversity showed no significant difference between healthy and infected plants. For fungal communities, there was no significant difference between treatments. According to PRRMANOVA, PVY and compartments exerted significant effects on bacterial and fungal community diversity indices (including H, S, and J values) (*p* < 0.05) (Table 2). Moreover, the effects of PVY and compartments were higher on bacterial communities than on fungal communities, as indicated by higher *R*^2^ values. In addition, the species richness of bacterial and fungal communities was influenced by the combination of field type and PVY. Significant tests based on MRPP, ANOSIM, and ADONIS were performed to analyze changes in microbial community structures using Bray–Curtis distance. The results suggested that the infection of disease caused significant changes in the endophytic bacterial community structure of plant stems and leaves in flooded fields. PERMANOVA analysis further revealed that the greatest effect on total microbial communities is by habitat (*R*^*2*^ = 0.244 for bacterial community and *R*^*2*^ = 0.272 for fungal community, *p* < 0.001), followed by PVY (*R*^*2*^ = 0.049 for bacterial community and *R*^*2*^ = 0.095 for fungal community, *p* < 0.001), and field type (*R*^*2*^ = 0.035 for bacterial community and *R*^*2*^ = 0.032 for fungal community, *p* < 0.001) (Table 3). Habitat and PVY explained a higher proportion of the variation in fungal community structure than that of bacterial community structure.

**Table 2:** Effects of field type, PVY, and plant compartments on bacterial and fungal community diversity indexes (*H*, Shannon Index; *S*, Species Richness; *J*, Pielous’ evenness) based on pairwise permutational multivariate analysis of variance (PERMANOVA) tests. ANOVA’s *R*^2^ values from the multivariable test were presented, and bold numbers meant the statistical significance (*p* < 0.05)

	Bacterial community			Fungal community		
	*H*	*S*	*J*	*H*	*S*	*J*
Group 1: field type	0.008	0.003	0.020	0.005	**0.003**	0.004
Group 2: PVY	**0.181**	**0.202**	**0.121**	**0.154**	**0.249**	0.033
Group 3: compartment	**0.513**	**0.701**	**0.318**	**0.197**	**0.669**	**0.131**
Group 1 × group 2	0.003	0.001	0.008	0.011	0.002	0.015
Group 1 × group 3	0.015	**0.013**	0.018	0.015	**0.007**	0.012
Group 2 × group 3	0.009	0.003	0.014	0.010	**0.007**	0.031
Group 1 × group 2 × group 3	0.012	0.002	0.022	0.012	0.001	0.008

**Table 3:** Pairwise permutational multivariate analysis of variance (PERMANOVA) showing the effect of field type, PVY, and plant compartment on bacterial and fungal community structures based on the Bray–Curtis distance metrics. ANOVA’s *R*^2^ and *p* values from the multivariable test were presented, and bold numbers meant the statistical significance (*p* < 0.05)

	Bacterial community			Fungal community	
	*R*^2^	*p*		*R*^2^	*p*
Group 1: field type	**0.035**	**0.000**	**0.032**		**0.000**
Group 2: PVY	**0.049**	**0.000**	**0.095**		**0.000**
Group 3: compartment	**0.244**	**0.000**	**0.272**		**0.000**
Group 1 × group 2	**0.022**	**0.006**	**0.020**		**0.046**
Group 1 × group 3	**0.070**	**0.000**	**0.051**		**0.000**
Group 2 × group 3	0.017	0.492	0.016		0.439
Group 1 × group 2 × group 3	0.018	0.399	0.012		0.854

### PVY shifted the plant microbial community compositions

**Fig. 1:**
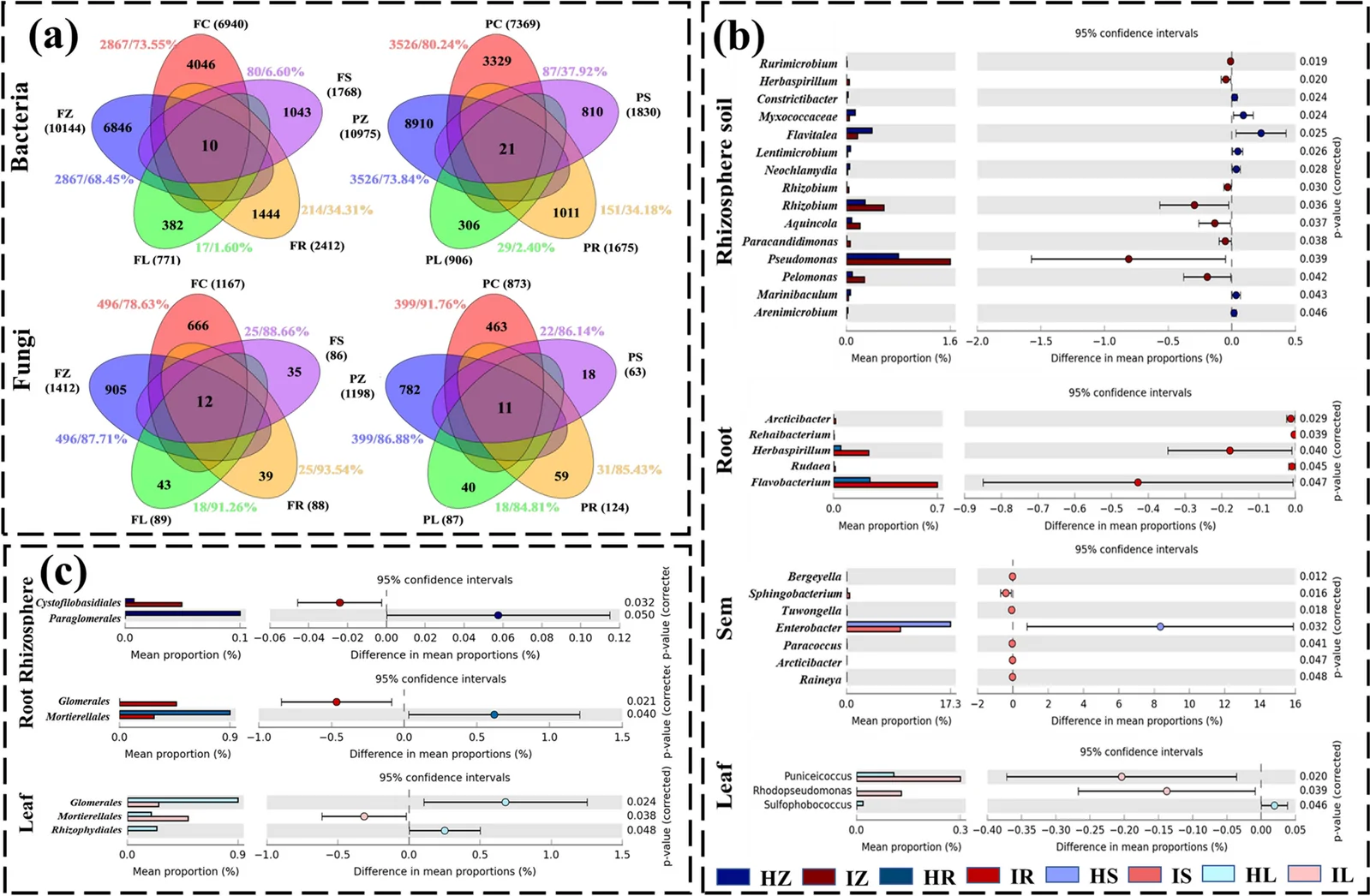
The enrichment and depletion patterns of bacterial and fungal microbial communities under PVY infection. **a** Venn diagram illustrated the overlap of bacterial and fungal communities in flooded field (F) and paddy field (P). The total number of ASVs were presented with black letter, and color number indicated the number and abundance of ASVs which was shared by field and rhizosphere soils in different plant compartments. Read for bulk soil (C), blue for rhizosphere soil (Z), green for plant leaf (L), yellow for root (R), and purple for stem (S). **b** Post hoc plot showing the differences of bacterial community composition at genus level between health and infected plant in different plant compartments (including rhizosphere soil, root, stem, and leaf). **c** Post hoc plot showing the differences of fungal community composition at order level between health and infected plant in different plant compartment. The differences between health and infected plant were observed by *t*-test, and significant difference indicated *p* < 0.05

The distributions of ASVs among bulk soil, rhizosphere soil, root, stem, and leaf were described by a Venn diagram (Fig. 1a). The results indicated that the total number of ASVs in rhizosphere and bulk soils was much higher than that in plant roots, stems, and leaves. For bacterial communities, the numbers of ASVs showed an obvious decreasing trend with the ascending migration from underground to aboveground. Specifically, rhizosphere bacteria had the highest numbers (10,144 for FZ and 109,751 for PZ), followed by endophytic bacterial communities in roots (2412 for FR and 1675 for PR), stems (1768 for FS and 1830 for PS), and leaves (771 for FL and 906 for PL). Moreover, the number and abundance of ASVs shared by field and rhizosphere soils also decreased with the ascending migration in flooded fields (FC 2867/73.55%, FZ 2867/68.45%, FR 214/34.31%, FS 80/6.60%, FL 17/1.60%) and paddy fields (PC 3526/80.24%, PZ 3526/73.84%, PR 151/34.18%, PS 87/37.92%, PL 29/2.40%). For fungal communities, the numbers of ASVs shared by field and rhizosphere continued to decrease with the ascending migration, while their abundance in endophytic communities (88.66–93.54% in flooded fields and 84.81–86.14% in paddy fields) negligibly differed from that in soil fungal communities.

Community composition analysis found that *Proteobacteria*, *Actinobacteria*, *Bacteroidetes*, *Firmicutes*, and *Chloroflexi* were dominant phyla for rhizosphere and endophytic bacterial communities (Fig. 1b, c). Student’s *T*-test analysis between healthy and infected plant bacterial communities showed that PVY infection significantly altered bacterial communities of rhizosphere soil, plant roots, plant stems, and plant leaves at the genus level. Specifically, 15 genera were significantly changed in the rhizosphere bacterial community, and the relative abundance of *Flavitalea*, *Myxococcaceae*, *Constrictibacter*, *Lentimicrobium*, *Neochlamydia*, *Marinibaculum*, and *Arenimicrobium* was significantly higher in healthy plants. In the root endophytic bacterial community, five genera significantly decreased under infection. In the stem endophytic bacterial community, seven genera were significantly changed, with *Enterobacter* enriched in the healthy plants. In the leaf endophytic bacterial community, three genera showed significant changes, with *Sulfophobococcus* enriched in healthy plants. In summary, PVY infection changed the bacterial communities of rhizosphere soil, plant roots, plant stems, and plant leaves at genus level, and the number of genera decreased with ascending migration. The dominant fungi were *Ascomycota*, *Basidiomycota*, *Chytridiomycota*, *Glomeromycota*, and *Zygomycota* in different treatments. Different results for the effect of infection on fungal community composition were found by *T*-test analysis compared to bacterial communities. In the rhizosphere fungal community, the relative abundance of two orders significantly changed, with *Paraglomerales* being enriched in the healthy plants. Similarly, in the root endophytic fungal community, two orders also showed significant changes, with *Mortierellales* being enriched in healthy plants.

*Contributions of biotic and abiotic factors on microbial community under PVY infection.*

**Fig. 2:**
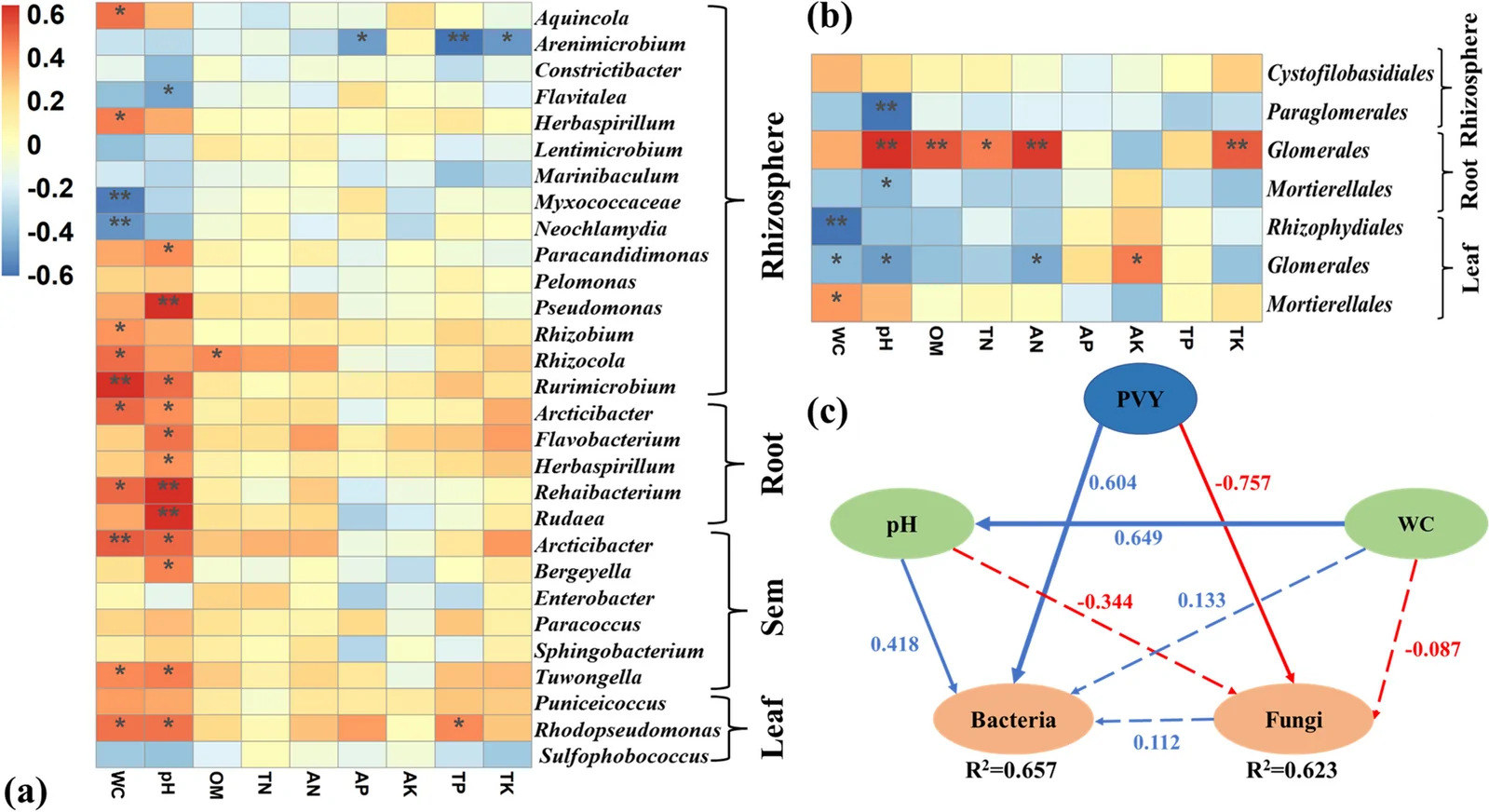
Heatmap demonstrated the correlation between soil properties and detected bacterial genera (**a**) and fungal orders (**b**) which were enriched or decreased under PVY infection, in rhizosphere soil, plant root, stem and leaf. **c** Partial least squares path model for the relationships between bacteria, fungi, soil properties (pH and WC) and PVY. Path coefficients were calculated and reflected in the width of the arrow, with read indicted negative effects and blue indicated positive effects. Solid arrows showed significant effects (*p* < 0.05). GoF value of the model was 0.520

In order to reveal the mechanism by which environmental factors regulate the core taxa of the microbial community, a spearman correlation analysis was conducted (Fig. 2). The results suggested that water content and pH played significant roles in the bacterial community. In addition, the core genera of rhizosphere and endophytic bacterial community showed significant relationships with rhizosphere soil water content (Fig. 2a), while soil pH showed significant correlations with endophytic bacterial community, especially in root tissue. Interestingly, nutrient contents including AP, TP, and TK were negatively correlated with *Arenimicrobium*, which was the core genus in the rhizosphere bacterial community. Spearman correlation analysis was also performed between the seven core orders in fungal communities and environmental factors (Fig. 2b). Similar to the results of bacterial communities, water content and pH were also critical environmental factors for fungal communities. However, the water content was positively correlated with *Mortierellales* and negatively correlated with *Glomeromycota* and *Rhizosphydiales*. Moreover, they were all core orders of the rhizosphere fungal community. The value of pH showed a significant correlation with the core orders of rhizosphere and endophytic fungal communities.

As discussed above, multi-parameters (i.e., environmental factors and PVY) affected the variations of microbial community. To better integrate the complex interrelationships between PVY, soil properties, specific bacterial, and fungal groups, a PLS-PM model was constructed (Fig. 2c). After model optimization, the GoF value was 0.520. Results suggested that factors including PVY, pH, and WC had a higher explanation for bacterial community (*R*^2^ = 0.657) than fungal community (*R*^2^ = 0.623). PVY showed positive and negative direct effects on bacterial (0.604) and fungal (− 0.757) communities, respectively. In addition, pH positively regulated the bacterial community (0.418), and WC had no direct effects on both bacterial and fungal communities. However, WC positively affected pH (0.649), which meant WC would indirectly affect bacterial community. Overall, PVY showed a larger effect on bacterial communities than fungal communities, and soil properties also affected bacterial and fungal communities.

### PVY affected plant bacterial and fungal networks

**Fig. 3:**
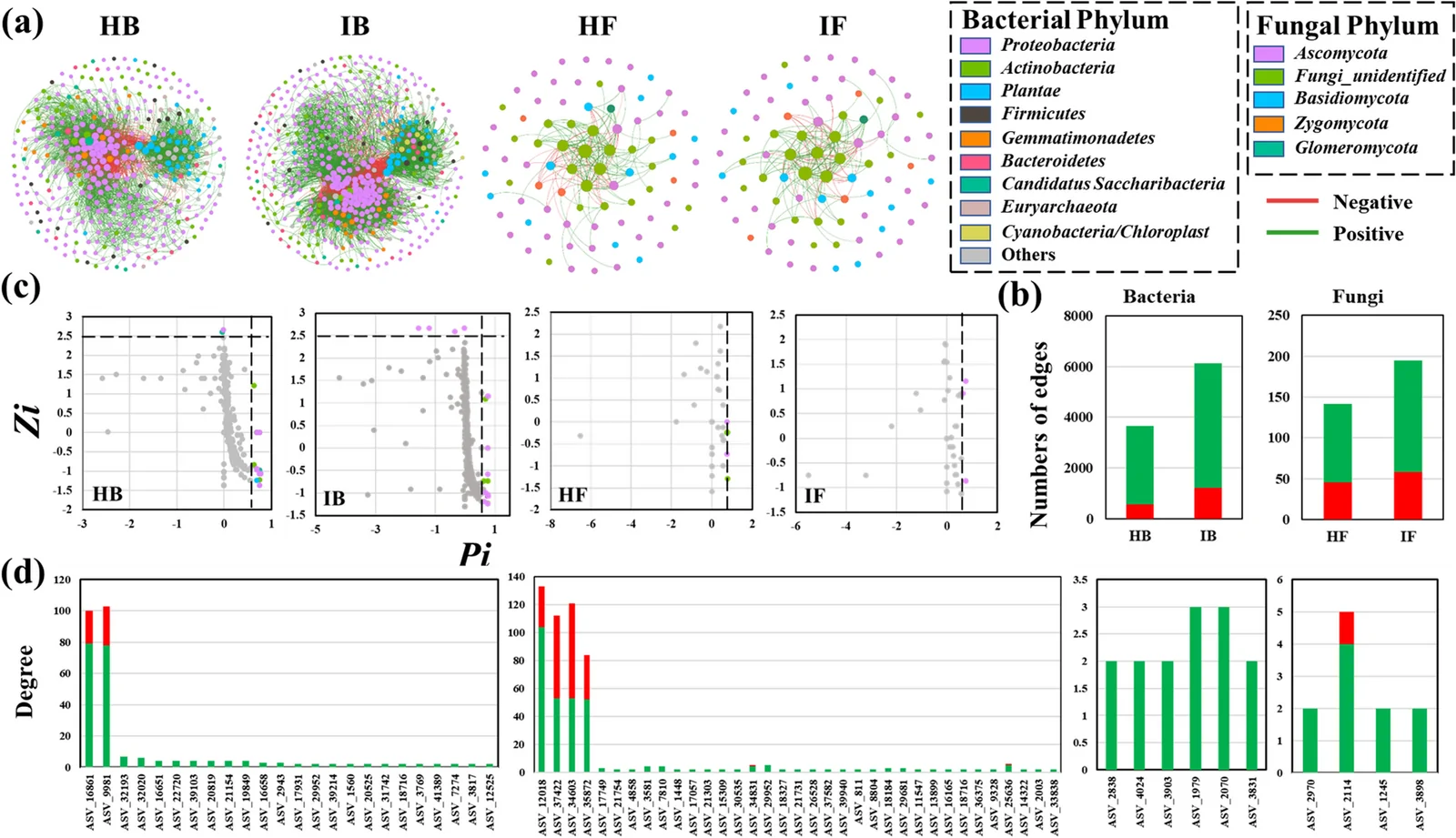
Bacterial and fungal network for health and infected plant. **a** The network showing a higher number of nodes and edges in bacterial networks (HB, IB) compared to fungal networks. The nodes were colored according to bacterial and fungal phylum, and sized by degree value. Positive edges were green, and negative edges were red. **b** Numbers of edges in bacterial and fungal community networks. **c** *Zi-Pi* plots identified the keystones in networks. Peripherals were gray points with *Zi* < 2.5 and *Pi* < 0.62. Module hubs (*Zi* ≥ 2.5 and *Pi* < 0.62), network hubs (*Zi* ≥ 2.5 and *Pi* ≥ 0.62), and connectors (*Zi* < 2.5 and *Pi* ≥ 0.62) were colored according to bacterial and fungal phylum. **d** Degree and edge type of the keystones in four networks

To investigate how PVY affected the plant microbiome, bacterial and fungal networks of both healthy and infected plants were constructed (Fig. 3a). The results showed that bacterial networks had a higher number of nodes and edges, modularity, and positive edge proportions (nodes/edges/modularity/proportion, 389/3647/0.452/84.45% in healthy plants, and 496/6133/0.402/80.14% in infected plants) than fungal networks (nodes/edges/modularity/proportion, 83/142/0.192/67.61% in healthy plants, and 100/195/0.275/70.26% in infected plants) (Fig. 3b). Further, there were 24, 35, 6, and 4 keystones (including module hubs, network hubs, and connectors) identified in HB, IB, HF, and IF networks, respectively (Fig. 3c). However, all the keystones in fungal networks were connectors. Compared to healthy plants networks, the number of keystones increased in infected plants’ bacterial networks and decreased in infected plants’ fungal networks. Keystones were primarily positive with other nodes but showed more negative correlations in fungal networks (Fig. 3d). Furthermore, several bacterial taxa, such as *Flavobacterium*, *Pseudomonas*, and *Sphingobacterium* enriched in the diseased plants, were also identified as keystones in IB network (Table S3). For fungal communities, the keystones were classified into *Cantharellales* order in HF network, while they were classified into *Sordariales* and *Hypocreales* orders in IF network (Table S4). In addition, bacterial and fungal networks in the infected plants were more complex (in terms of the number of nodes, edges, average degree, and average path length) than those in the healthy plants.

## Discussion

In this study, we sought to investigate the effects of PVY on plant microbiomes using amplicon sequencing approaches. By profiling both bacterial and fungal communities in below- and aboveground compartments of healthy and PVY-infected plants, we revealed that bacterial networks were more complex and their communities were more sensitive to PVY than fungal communities. pH and WC played important roles in shaping microbial community composition as key soil properties. Moreover, our work found that bacterial and fungal communities in plant organs had different recruitment strategies. Through this work, we have provided evidence that PVY infection not only changed the diversity and composition of microbial communities but also influenced their networks. Below, we discussed how these findings promoted our understanding of disease-induced changes in plant microbial communities and ecological networks.

### Bacterial and fungal communities showed different recruitment strategies for plant organs

Uncovering how microbes were recruited to plant endophytic communities is of great importance to advance the microbial assembly of plants during disease infection. Accumulating studies on wheat, sugar beet, and *Arabidopsis thaliana* have found that plant roots could attract beneficial species to resist pathogen infection, which was called as “cry for help” strategy (Berendsen et al. 2018; Carrión et al. 2019; Yin et al. 2021). Plants are hosts of complex endophytic microbial communities, which could colonize both below- and aboveground tissues. Endophytes could be recruited from the surrounding environment horizontally or transmitted through seeds vertically. Colonization of endophytes in roots from soil is the most important transmission route (Frank et al. 2017). Rhizosphere microbial communities were recruited by plants based on carbon sources, phytochemicals, pH, oxygen, and root exudates, acting as a selective barrier to plant roots. However, microbial communities had a higher number in rhizosphere soil than in bulk soil in our study. We speculate that the complex substances (e.g., amino acids, sugars, organic acids, mucilage, and proteins) secreted from plant roots were not only used to select special taxa, but also to increase soil nutrition in rhizoplanes, such as carbon sources, which would help to increase the rhizosphere microbial community diversity (Zhang et al. 2017). With the selection pressure from plants, the microbial community drastically decreased in plant tissues compared to that in soils. Interestingly, the ASV numbers of endophytic bacterial communities in root, stem, and leaf were gradually reduced, as well as the number and abundance of ASVs shared in rhizosphere and bulk soils; however, the number and abundance of ASVs had no obvious changes in endophytic fungal communities. Different transmission strategies for endophytes between bacteria and fungi were caused by different sources of species. Endophytic fungi are usually derived from aerial fungal spores, while most endophytic bacteria are derived from rhizosphere soil (Wang et al. 2016). In addition, changes in field types had no influence on the transmission strategies of bacterial and fungal communities.

Understanding the keystone species through the analysis of network hubs and their associations with other species is crucial for leveraging the plant microbiome to improve plant growth and health (Gao et al. 2021). This study identified *Flavobacterium*, *Pseudomonas*, and *Sphingobacterium* as potential beneficial bacteria and network keystones in plant microbiomes, which were enriched in diseased plants. Prior research has demonstrated the presence of numerous members from the *Flavobacterium*, *Pseudomonas*, and *Sphingobacterium* genera in various plant compartments, highlighting their significant role in influencing host performance, especially in plant pathogens (Etesami and Adl 2020). For instance, *Pseudomonas* emerges as the prevailing taxon among plant-beneficial bacteria, playing a crucial role in safeguarding plants against pathogens (Yu et al. 2019). Moreover, network keystones assume pivotal topological positions and may be deployed to organize favorable plant microbiomes. The convergence observed between biomarker taxa and network keystones also implies that certain bacterial taxa recruited by diseased plants may function as keystone species within plant microbiomes, thereby ensuring the perpetuation of future generations.

### Bacterial communities were more sensitive to PVY than fungal communities

Cooperative and competitive interactions between microbial species and network topological properties played important roles in community stability. In this study, bacterial networks, including healthy and infected plants, were characterized by a higher proportion of positive correlations than those in fungal networks. Positive correlations in networks mean ecological cooperation between species, which creates dependency and potential for mutual downfall (Coyte et al. 2015). Thus, a higher proportion of positive correlations in bacterial networks indicated lower microbiome stability compared to fungal networks. Higher competition in fungal networks would provide more resistance to external stress, such as disease infection (Wagg et al. 2019). In contrast to fungal communities, bacterial communities were more affected by PVY, due to increased negative correlations between bacterial species in PVY-infected networks compared to healthy networks. In addition, higher modularity and complexity (as indicated by the number of nodes, edges, average degree, and average path length) were observed in healthy bacterial networks, further exacerbating the destabilizing effect. Higher modularity means higher prevalence of cross-module correlations among taxa (Grilli et al. 2016). Network complexity is strongly correlated with network stability, which supports the central ecological theory that complexity leads to stability (Yuan et al. 2021). These findings suggested that bacterial communities were more sensitive to PVY than fungal communities, and infection increased community stability. Studies have found that the bacterial community was less stable than the fungal community under environmental disturbances, such as drought stress (de Vries et al. 2018b) and manure application (Wang et al. 2021). However, a previous study reported that the fungal community was more sensitive to *Fusarium* wilt disease (FWD) than the bacterial community (Gao et al. 2021). The possible reason for this discrepancy was that the networks were based on plant roots, stems, and fruits, where the disease affected the microbial community of reproductive organs less than vegetative organs. In our study, however, the network included soil, root, stem, and leaf samples, which caused the contrasting results.

Our study indicated that PVY increased bacterial network stability and decreased fungal network stability. The contrasting pattern between bacterial and fungal networks was observed based on network complexity properties such as average degree and average path length. Previous studies have reported that network complexity and keystones are both important for community stability (Toju et al. 2018; Wagg et al. 2019). PVY decreased the number of connectors in fungal networks, but increased the number of module hubs in bacterial networks. Moreover, PVY increased competition between keystones and other species. Bacterial networks showed higher modularity, and more competition was established between species within the module responding to disease infection. Conversely, the correlation between modules of fungal networks was decreased, and competition between connectors and other species was increased. Indeed, the negative effect of *V. dahliae* (a kind of soilborne pathogen) has been demonstrated to be alleviated by decreasing the correlations between modules (Rybakova et al. 2017).

In this study, samples included variations in field type, PVY, and compartment. Analysis of bacterial and fungal community diversity found that PVY and compartment had a higher impact on the fungal community, while field type showed higher effects on the bacterial community. Different field types implied different soil characteristics (e.g., WC, pH, AHN, and TK), which affected microbial community structure and diversity. Microbial communities in different compartments of a plant showed a high degree of organ specificity, with different selective pressures (Guevara-Araya et al. 2020). PVY has also been found to affect the assembly of microbial community, as a typical plant disease (Chowdhury et al. 2021; Gao et al. 2021). In addition, bacteria and fungi had different body sizes (Gu et al. 2022b), diversity, dispersal potential, ecological function (Gao et al. 2021), and correlation with host and other microorganisms, ultimately affecting the species sorting and community assembly. Higher effects of field type, PVY, and compartment on bacterial community diversity and structure meant that the bacterial community was more sensitive to environmental factors compared to fungal community.

### pH and WC drove microbial community assembly under PVY infection

Our results showed that bacterial and fungal community composition was significantly different between healthy and infected plants, with plant growth–promoting microbes enriched in rhizosphere soil, plant root, stem, and leaf. Previous studies have shown that plants lacking genetic resistance to pathogens would enrich particular microbes to obtain pathogen suppression (Santhanam et al. 2015). For bacterial communities, *Flavitalea*, *Myxococcaceae*, *Constrictibacter*, *Lentimicrobium*, *Neochlamydia*, *Marinibaculum*, *Arenimicrobium*, *Enterobacter*, and *Sulfophobococcus* were enriched in healthy plants. It has been reported that *Myxococcaceae* could produce antagonistic enzymes and secondary metabolites that maintain plant health (Dror et al. 2022), and *Enterobacter* was an endophytic plant growth–promoting bacterium (Taghavi et al. 2010). For fungal communities, *Paraglomerales*, *Mortierellales*, *Glomerales*, and *Rhizophydiales* were also enriched in healthy plants. *Paraglomerales* belonged to arbuscular mycorrhizal fungi, which provided essential nutrients to plants and improved plant health and production (Bano and Uzair 2021). Soil properties, especially for pH and water content, have been shown to play an important role in changes in microbial composition. Previous studies have found that the infection of plant pathogens could be significantly and directly regulated by soil properties, including pH (Li et al. 2017), temperature, water content (Jiang et al. 2021a), and resource availability (Yang et al. 2017). Soil pH has been reported to determine the colonization of plant pathogens by impacting the specific microbial groups (Liu et al. 2023). Water content of soil could affect the nutrient availability, determining the microbial community composition by increasing Gram-negative bacteria but decreasing Gram-positive bacteria and fungi (Chen et al. 2020). In our study, the water content showed negative and direct effects on bacterial and fungal communities, and pH had a positive influence on fungal communities directly. Considering the correlations between bacteria and fungi, the bacterial community was also indirectly affected by pH.

In this study, we found that bacterial and fungal communities showed different recruitment strategies in plant organs. The number and abundance of shared bacterial ASVs in bulk and rhizosphere soils decreased with ascending migration from below- to aboveground compartments, while the number and abundance of fungal ASVs showed no obvious changes. Field type, plant compartments, and PVY infection all affected microbial community diversity and structures, except for field type on microbial community diversity. In addition, rhizosphere soil pH and WC drove bacterial and fungal community assembly processes directly under PVY infection. Analysis of microbial networks indicated that bacterial communities were more sensitive to PVY than fungal communities, as evidenced by lower network stability of bacterial communities due to a higher proportion of positive edges. PVY infection further increased bacterial network stability, and decreased fungal network stability.

## Supplementary information

Below is the link to the electronic supplementary material.

Supplementary file1 (PDF 172 KB)

## Data Availability

The datasets generated during and/or analyzed during the current study are available in NCBI database, in the project of PRJNA946037 and PRJNA946055.
