# Supplementary material for: Disease-induced changes in bacterial and fungal communities from plant below- and aboveground compartments
Source: Appl Microbiol Biotechnol. 2024 Apr 30;108(1):315. doi: 10.1007/s00253-024-13150-1 (PMC11061026; doi:10.1007/s00253-024-13150-1)
Supplement: Supplementary file 1 — Supplementary file1 (PDF 172 KB) [file 253_2024_13150_MOESM1_ESM.pdf]

Applied Microbiology and Biotechnology

Disease-induced changes in bacterial and fungal communities from plant below- and aboveground compartments

Mingfeng Cao[a], Songqing Huang[a], Jingjing Li[c], Xiaoming Zhang[a], Yi Zhu[a], Jingzhao Sun[a], Li Zhu[a], Yong Deng[a], Jianqiang Xu[a], Zhihua Zhang[a], Qiang Li[a], Jixiang Ai[a], Tian Xie[a], Hengli Li[a], Huaqun Yin[b], **Wuyuan Kong[a*], Yabing Gu[b*]**

[a] Changde Tobacco Company of Hunan Province, Changde, China;

[b] School of Minerals Processing and Bioengineering, Central South University, Changsha, China;

[c] Technology Center of China Tobacco Fujian company, Xiamen, China.

* Corresponding author:

Wuyuan Kong: 809033168@qq.com;

Yabing Gu: 1466552951@qq.com.

Table 1. The diversity indices (H, Shannon Index; S, Species richness; J, Pielous' evenness) of bacterial communities in different treatments (mean value ± standard error).

**Treatment**	**H**	**S**	**J**
SCK	6.87±0.189	1586.8±148.4	0.933±0.0175
SH	6.83±0.294	1567.2±285.2	0.929±0.0184
SHL	3.42±0.544	124.0±32.8	0.710±0.0874
SHR	4.52±0.356	368.2±65.0	0.767±0.0449
SHS	3.83±0.956	249.5±79.4	0.696±0.1440
SI	6.78±0.280	1500.5±228.0	0.929±0.0195
SIL	3.71±0.918	147.5±30.9	0.740±0.1655
SIR	4.19±0.572	399.5±65.8	0.699±0.0831
SIS	4.61±0.279	319.5±38.4	0.801±0.0590
TCK	7.03±0.289	1882.7±268.6	0.933±0.0213
TH	6.86±0.197	1748.8±224.1	0.920±0.0114
THL	3.53±0.966	145.5±35.3	0.708±0.1750
THR	3.25±1.120	258.8±131.7	0.585±0.1618
THS	3.72±0.745	299.5±114.9	0.654±0.0903
TI	6.99±0.106	1827.7±157.2	0.931±0.0078
TIL	3.50±0.589	146.0±20.6	0.703±0.1152
TIR	3.90±0.430	301.2±70.3	0.685±0.0584
TIS	4.22±0.561	338.8±53.9	0.725±0.0842

Table 2. The diversity indices (H, Shannon Index; S, Species richness; J, Pielous' evenness) of fungal communities in different treatments (mean value ± standard error).

Treatment	H	S	J
SCK	3.26±0.650	284.2±42.0	0.577±0.1042
SH	2.62±0.878	202.5±59.3	0.491±0.1458
SHL	1.97±0.182	21.3±4.2	0.648±0.0358
SHR	1.72±0.189	16.0±3.2	0.625±0.0554
SHS	1.92±0.257	17.7±4.8	0.675±0.0349
SI	2.95±1.126	242.7±72.2	0.533±0.1983
SIL	1.91±0.287	17.7±4.2	0.668±0.0588
SIR	1.72±0.177	18.2±1.6	0.594±0.0552
SIS	1.79±0.213	20.8±5.1	0.599±0.0982
TCK	3.05±0.758	234.3±58.2	0.557±0.1153
TH	2.98±0.824	220.2±52.8	0.551±0.1428
THL	2.15±0.103	23.2±2.7	0.686±0.0284
THR	2.06±0.320	21.2±8.6	0.703±0.1652
THS	2.05±0.164	15.7±2.7	0.749±0.0506
TI	2.67±0.845	191.8±14.6	0.507±0.1581
TIL	2.05±0.158	21.3±3.4	0.675±0.0655
TIR	1.98±0.566	25.8±3.5	0.609±0.1669
TIS	1.62±0.759	19.5±4.1	0.560±0.2672

Table 3. The classication information and topological roles of keystone species in bacterial networks HB and IB.

Network	Genus	Id	Topological role
HB	*Acidovorax*	ASV_16658	Connectors
	Acinetobacter	ASV_29952	Connectors
	Arthrobacter	ASV_20525, ASV_41389	Connectors
	Chryseobacterium	ASV_20819	Connectors
	Enterobacter	ASV_19849, ASV_21154	Connectors
	Methylobacillus	ASV_16651	Connectors
	Methylobacterium	ASV_16861	Connectors
	Methylorubrum	ASV_18716	Connectors
	Niastella	ASV_22720	Connectors
	Pectobacterium	ASV_9981, ASV_39214	Connectors
	Pseudarthrobacter	ASV_17931, ASV_1560	Connectors
	Pseudomonas	ASV_32020, ASV_31742	Connectors
	Raoultella	ASV_32193	Connectors
	Roseateles	ASV_2943	Connectors
	Sphingomonas	ASV_3817	Module hubs
	Streptomyces	ASV_7274	Connectors
	Unclassified	ASV_39103	Connectors
		ASV_12525	Module hubs
	Zea	ASV_3769	Connectors
IB	*Acidovorax*	ASV_21303	Connectors
	Baekduia	ASV_811	Connectors
	Bradyrhizobium	ASV_12018	Module hubs
	Cellulomonas	ASV_11547, ASV_36375	Connectors
	Chryseobacterium	ASV_29681	Connectors
	Citrobacter	ASV_37422	Module hubs
	Ensifer	ASV_21731	Connectors

Enterobacter	ASV_34603	Module hubs
Enterococcus	ASV_18716	Connectors
Escherichia/Shigella	ASV_25636	Connectors
Flavobacterium	ASV_16165, ASV_9328	Connectors
Herminiimonas	ASV_18184	Connectors
Lentzea	ASV_39940	Connectors
Lysinimonas	ASV_1448	Connectors
Lysobacter	ASV_17749	Connectors
Methylophilus	ASV_17057	Connectors
Nocardioides	ASV_14322	Connectors
Pseudoduganella	ASV_4858	Connectors
Pseudomonas	ASV_29952	Connectors
Rhizorhapis	ASV_18327	Connectors
Sphingobacterium	ASV_13899	Connectors
Sphingopyxis	ASV_26528	Connectors
Stenotrophomonas	ASV_35872	Module hubs
Streptomyces	ASV_3581, ASV_7810, ASV_15309, ASV_30535, ASV_34831, ASV_37582	Connectors
Unclassified	ASV_8804, ASV_33838	Connectors
Variovorax	ASV_21754	Connectors
Vespertiliibacter	ASV_2003	Connectors

Table 4. The classication information and topological roles of keystone species in fungal networks HF and IF.

Network	Order	Id	Topological role
HF	*Cantharellales*	ASV_3903	Connectors
	Unclassified	ASV_1979, ASV_2070, ASV_3831, ASV_2838, ASV_4024	Connectors
IF	*Sordariales*	ASV_2114	Connectors
	Hypocreales	ASV_3898	Connectors
	Unclassified	ASV_1245, ASV_2970	Connectors
